# Temporal Sensory Profiles of Regular and Sodium-Reduced Foods Elicited by Temporal Dominance of Sensations (TDS) and Temporal Check-All-That-Apply (TCATA)

**DOI:** 10.3390/foods11030457

**Published:** 2022-02-03

**Authors:** Ha Nguyen, Wendy V. Wismer

**Affiliations:** Department of Agricultural, Food and Nutritional Science, University of Alberta, Edmonton, AB T6G 2P5, Canada; ntt.ha84@hutech.edu.vn

**Keywords:** sodium reduction, temporal sensory profiles, Temporal Dominance of Sensation (TDS), Temporal-Check-All-That-Apply (TCATA), multiple intake

## Abstract

Temporal sensory methods can be used to highlight the impact of sodium reduction on the dynamic sensory profile of foods targeted for sodium reduction. Study aims were to compare the temporal sensory attribute profiles of regular and sodium-reduced food products elicited by TDS and TCATA, over single and multiple oral intakes. A total of 20 semi-trained participants evaluated commercially available regular and sodium-reduced canned corn, cooked ham (single intakes), potato chips and cream of mushroom soup (5 intakes) using both TDS and TCATA. Regular and sodium-reduced products differed in not only *salty* but also other sensory attributes, noticeably *dry* for chips, *sweet* for corn, *bitter* and *metallic* for ham, *thick*, *creamy*, *sweet*, and *starchy* for soup. TDS and TCATA provided comparable information for the key sensory attributes characterizing and differentiating the regular and sodium-reduced products. TDS profiled significant differences between samples for a larger number of attributes than TCATA, while TCATA profiles were more consistent across intakes. Multiple intakes changed the duration of attribute dominance but not the number of significantly dominant attributes in TDS profiles. The current findings provide insight for applications of temporal profiling to other food products and development of sodium-reduced foods with attribute profiles acceptable to consumers.

## 1. Introduction

Excessive sodium intake has been linked to negative health consequences [1,2]. The majority of dietary sodium in high and upper-middle income countries, including Canada [3] and the United States [4], comes from packaged and prepared foods such as bread and processed cereal products, processed meats, cheese, salty snack foods, condiments and soups [1,5,6,7].

Globally, the food industry has made efforts to reduce sodium levels in processed food products [8] using a range of strategies, including gradual sodium reduction, the use of salt substitutes or flavour enhancers, changes in the morphology and size of salt crystals and improvement of salt diffusion by modification of the food matrix [9,10,11]. However, the commercial success of sodium-reduced food products is limited as sodium reduction not only affects salty taste but also other product sensory attributes [9,12,13] that are important contributors to consumer preference and satisfaction with foods [14].

Food sensory perception is a dynamic phenomenon in which perceived sensory attributes change with the in-mouth transformation of the food [15]. Sensory data collected using static methods, in which assessors rate the perceived intensity of each attribute only once throughout the evaluation, may omit relevant information to understand consumer preferences [16]. Most previous studies involving sodium-reduced food products have used static approaches for the sensory characterization of products rather than dynamic methodologies. However, in the last decade, an increasing number of studies have addressed the effect of salt reduction on the temporal sensory profile of different food categories such as sausages and ham, cheese, cream cheese, butter, margarine, bread and shoestring potatoes [17,18,19,20,21,22,23,24,25,26,27,28]. Temporal sensory methods have proven useful for understanding the impact of sodium reduction during reformulation processes [10].

Among the current temporal sensory profiling methods [29], Time Intensity (TI), Temporal Dominance of Sensations (TDS) and Temporal Check-All-That-Apply (TCATA) have been applied to the evaluation of sodium-reduced products. Temporal changes in multiple sensory attributes can be concurrently evaluated by TDS and TCATA. TDS was originally proposed as a multi-attribute temporal sensory method that scaled the intensities of a sequence of dominant attributes [30]. A variant of TDS [31], in which the dominant attribute is selected without scaling its intensity, has been widely used. TCATA is a more recent temporal method used to generate a continuous description of the sensory characteristics concurrently perceived in products [28,32]. TDS and TCATA have been compared across several product categories [33,34,35,36,37,38,39,40], but with limited application to regular and sodium-reduced foods [22,41]. The application of these temporal methods to highlight the impact of sodium reduction on the dynamic sensory profile of different product categories is warranted.

Additionally, multiple intake evaluations are thought to better reflect the sensory experience during real consumption settings than single-intake assessments. Previous studies reported significant differences in the TDS profiles among intakes for beer [42], wine and cheese [43,44,45,46], milkshake [47] and orange juice [48]. Multi-sip/bite evaluations may provide greater insight of the differences between regular and sodium-reduced product temporal profiles.

The objectives of this study were (1) to compare temporal sensory profiles between commonly consumed salty food products available in the marketplace and their sodium-reduced counterparts, and (2) to compare the TDS and TCATA temporal sensory profiles of these products, over single and multiple intakes.

## 2. Materials and Methods

***Participants.*** Participants were recruited at the University of Alberta (Edmonton, Canada) and were 18+ years of age and regular consumers of the study products. Exclusion criteria included smoking, pregnancy or having a thyroid condition. Interested participants (n = 45) completed a series of sensory acuity screening tests including basic taste identification, odor identification, food product description and PROP taster status, in a 20-minute session [49]. A total of 20 participants (15 females, 18–49 years) were selected for the panel based on the screening test results. The study protocol was approved by a Research Ethics Board at the University of Alberta. Participants completed written informed consent and received a gift card in acknowledgement of their time and contribution to the study.

***Food samples and preparation.*** Four pairs of commonly consumed food products were selected to represent a range of regular and sodium-reduced processed foods available in the Canadian marketplace: potato chips, cooked ham, canned corn, and cream of mushroom soup. Product information, sodium content and the amount of sample assessors received for each product evaluation are presented in Table 1. Moreover, 5 potato chips, 1 chip for each intake, were served in 162 mL clear plastic cups with lids. The hams were sliced into 0.3 × 2 × 4 (cm) pieces and served in a 162 mL clear plastic cup with a lid. Canned corn was heated, and 10 corn kernels were served in a 50 mL clear jar with a lid. Soup samples were prepared with 2% partly skimmed milk following the manufacturer’s instruction, and 60 mL of soup were served in 125 mL clear jars with lids (a 10 mL spoon was used for each soup bite evaluation). Corn and soup samples were kept at 60 °C in a water bath before serving. Chips and ham were served at room temperature. All samples were blinded with random 3-digit codes. Distilled water and unsalted crackers were provided to cleanse the palate between samples.

***Temporal sensory characterization of regular and sodium-reduced products.*** Participants attended 6 1-h sessions to generate the lists of attributes, standardize the tasting protocol and become familiar with the use of Compusense Cloud (Compusense, Inc., Guelph, ON, Canada) on a tablet to generate TDS [30] and TCATA [32] temporal sensory profiles. For TDS, assessors indicated the dominant sensation in the sample at each moment [30]. For TCATA, assessors checked the listed terms to describe the sensory attributes perceived in the sample at each moment. TCATA fading with an 8.0 s fade time [28] was used, thus it was not necessary to uncheck term.

Food product attribute lists included flavour and texture descriptors (Table 2) and were identical for TDS and TCATA evaluations. The number of attributes ranged from 8 to 12, in line with literature recommendations [29,31,50]. Participants placed the entire sample in the mouth and evaluated it for up to 60 seconds; they could stop the evaluation prior to this timepoint if no sensation was perceived. Swallowing timepoints were used as a reminder to swallow the sample according to time defined by consensus in the standardization of the tasting protocol (Table 1) [16,29]. Participants were instructed to chew the sample as usual and swallow when the message “Swallow” displayed on the screen. Corn and ham were assessed by single-intake evaluation while multi-intake evaluation was used for chips (5 bites) and soup (5 sips).

A within-subject experimental design was used; each participant assessed the temporal sensory profiles of the two samples of each product in triplicate. TDS and TCATA evaluations were completed on different days with randomized orders among participants; two study samples and one warm-up sample for each product were evaluated on each day. Participants waited at least 1 minute between the samples and took a 5-minute break between products. The order of attributes in the attribute list, the sample orders for each product and the orders of product types were balanced among participants using Williams’ Latin square designs.

***Data analysis.*** To remove individual differences in mastication rates, the data from each participant in each replicate (judgement) was standardized according to individual mastication durations; timing began when the first attribute was selected [51]. TDS data were analyzed as recommended by Pineau et al. (2009). TCATA data were analyzed as recommended by Castura et al. (2016). All data analyses were carried out using R version 3.4.1 [52]. TDS and TCATA curves (dominance rates against time for TDS and citation proportions against time for TCATA, respectively), and product trajectories for multi-intake evaluations of chips and soup were plotted using the *tempR* package [53]. Difference curves for pairs of regular and low sodium products were obtained by subtracting their dominance rates or citation proportions. A Fisher exact test was applied at each time point to determine statistical significance from zero (*p* ≤ 0.05), and significant difference curves were plotted. To obtain product trajectories, Principal Component Analysis (PCA) was conducted on data frames of mean citation proportions (TCATA) or dominance rates (TDS), in which each row is a Product*Time and each column is an Attribute [53].

## 3. Results and Discussion

### 3.1. Temporal Sensory Profiling of Regular and Sodium-Reduced Foods

**Figure 1:**
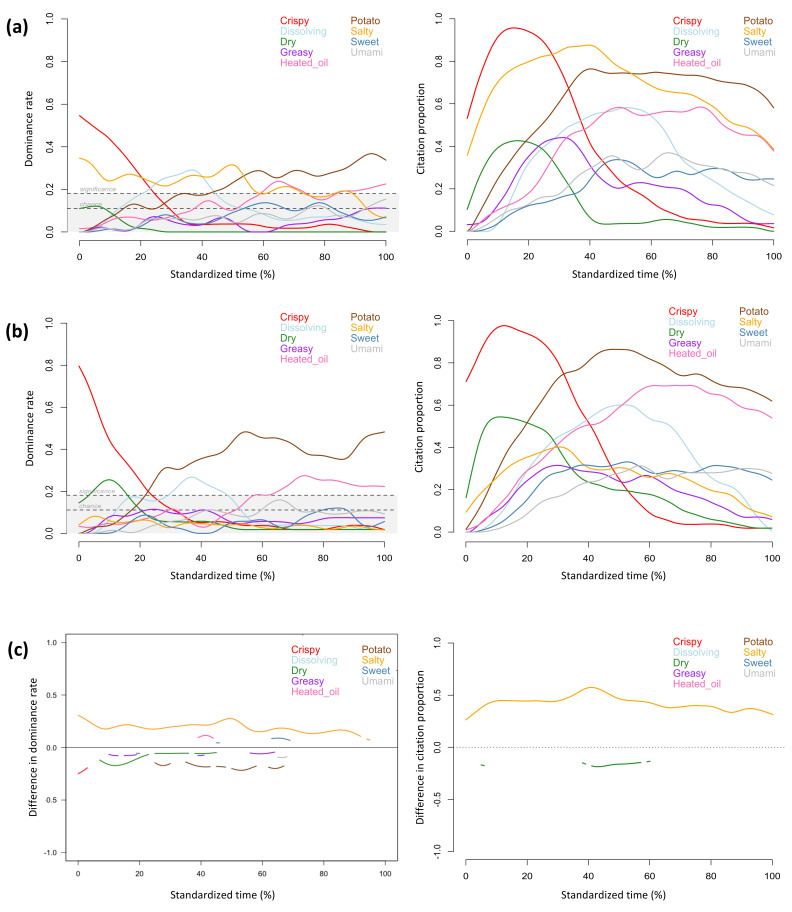
Temporal profiles for regular (**a**) and sodium-reduced (**b**) chips, and significant difference curves (**c**) obtained by TDS (left) and TCATA (right) evaluations of the first intake (n = 54).

**Figure 2:**
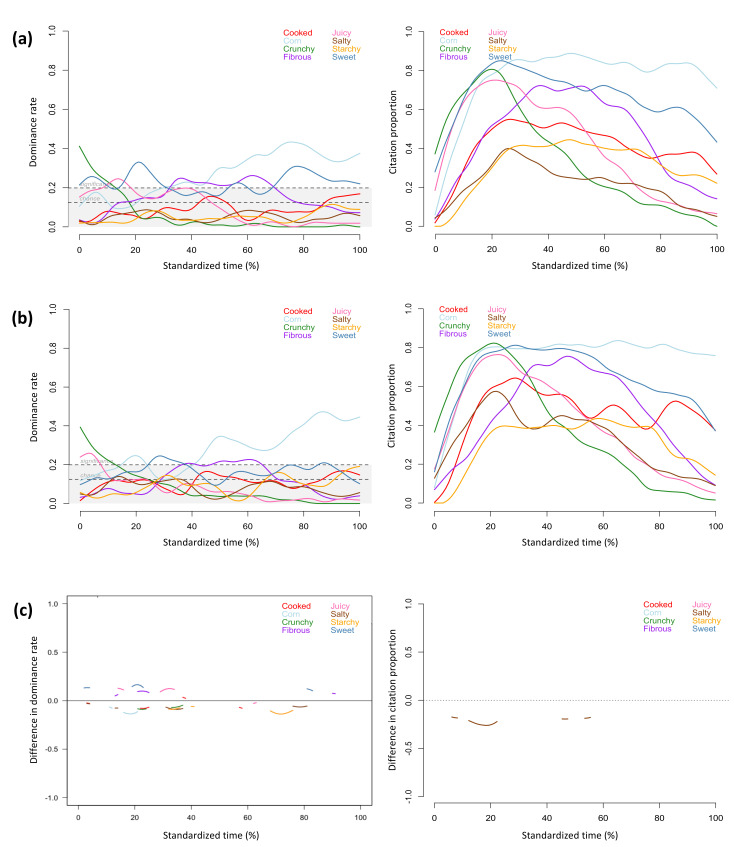
Temporal profiles for regular (**a**) and sodium-reduced (**b**) corn, and significant difference curves (**c**) obtained by TDS (left) and TCATA (right) (n = 54).

**Figure 3:**
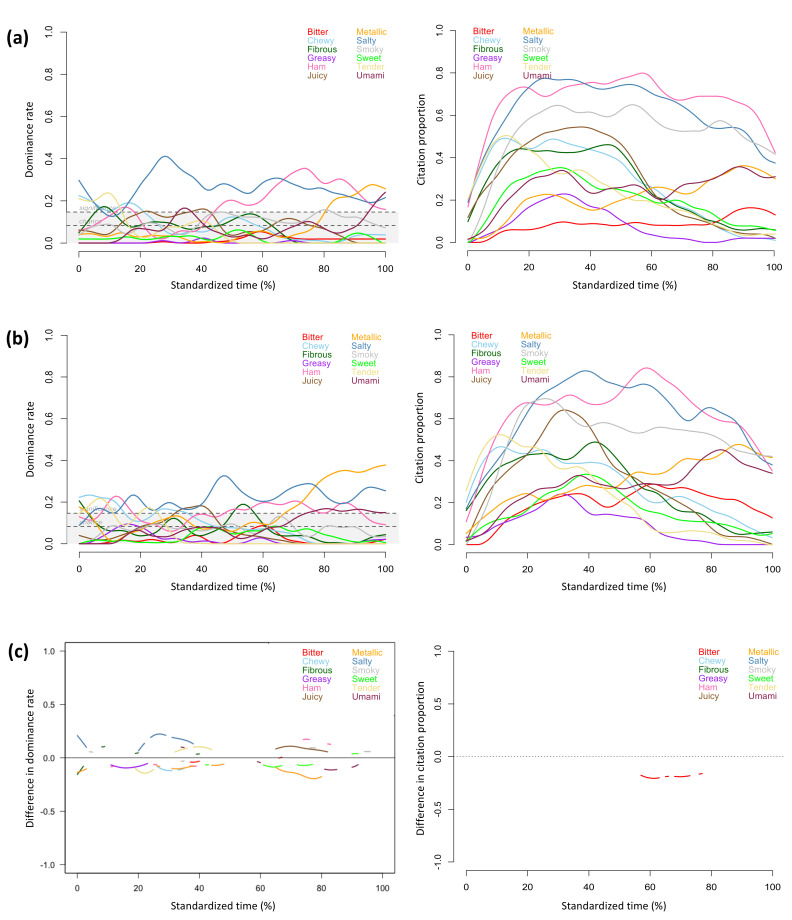
Temporal profiles for regular (**a**) and sodium-reduced (**b**) ham, and significant difference curves (**c**) obtained by TDS (left) and TCATA (right) (n = 51).

**Figure 4:**
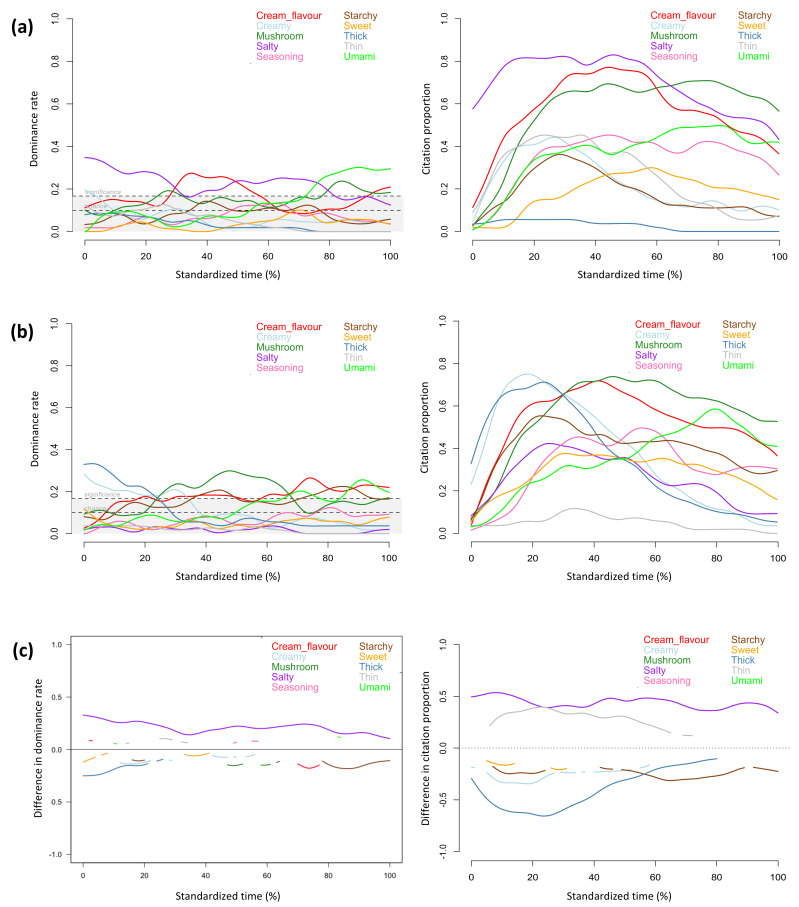
Temporal profiles for regular (**a**) and sodium-reduced (**b**) soup, and significant difference curves (**c**) obtained by TDS (left) and TCATA (right) evaluations of the first intake (n = 54).

The TDS and TCATA profiles provided similar information about the sensory attributes that characterized the regular and the sodium-reduced products throughout the evaluation period. The attributes reaching significant dominance rates in TDS profiles also showed high citation proportions in TCATA profiles, including *crispy, dissolving, salty, potato,* and *heated oil* for the regular chips, *crispy, dry, dissolving, potato,* and *heated oil* for the sodium-reduced chips (Figure 1a,b), *crunchy, juicy*, *fibrous, corn* and *sweet* for the regular and sodium-reduced corn (Figure 2a,b), *chewy, tender, fibrous, juicy, salty, ham, umami,* and *metallic* for the regular and sodium-reduced ham (Figure 3a,b), *salty*, *cream flavour*, *mushroom*, and *umami* for the regular soup, and *thick*, *creamy*, *mushroom*, *cream flavour*, *starchy,* and *umami* for the sodium-reduced soup (Figure 4a,b).

Texture attributes characterized products at the beginning of evaluation (i.e., *crispy* for chips, *dry* for sodium-reduced chips, *crunchy* and *juicy* for corn, *chewy* and *tender* for ham, and *thick* and *creamy* for sodium-reduced soup), and flavour attributes became more prominent during the chewing process, which aligns with previous studies on TDS evaluation of solid and semi-solid foods [22,54,55]. In the current study, this observation was consistent across both TDS and TCATA profiles of all of the foods evaluated.

However, some differences were observed between the two methods in attribute evolution during the evaluation period. For example, TDS and TCATA profiles of mushroom soup differed in the period of time during which the sensory attributes were relevant for characterizing samples (e.g., *thick, creamy, cream flavour, mushroom,* and *starchy*). *Mushroom* was highly cited during mid to late TCATA evaluation of both the regular and sodium-reduced soups, however, in the TDS evaluation this attribute evolved differently between the 2 soups (22–32% and 78–100% of the evaluation time for the regular soup, and 22–70% and several seconds at the end of the evaluation time for the sodium-reduced one). Similarly, the TDS and TCATA profiles of the sodium-reduced soup showed clear differences of *starchy* evolution throughout the evaluation period.

Aligning with observations of previous studies [22,33,36], TCATA provided more detailed description of concurrent sensory attributes at given timepoints. As only one attribute can be selected as dominant at evaluation time points during TDS evaluation, attribute curves more frequently fluctuated in TDS than TCATA profiles. For the regular chips (Figure 1a), *dissolving* and *heated oil* had comparable citation proportions near the mid evaluation in the TCATA profile, but only *dissolving* was significantly dominant at the same timepoint in the TDS profile and even reached the highest dominance rate within 25% to 40% of the evaluation time. *Cooked* for the regular corn and *cooked* and *salty* for the sodium-reduced corn reached citation proportions of more than 50% in the TCATA evaluation but were not significantly dominant in TDS (Figure 2a,b). For the sodium-reduced corn, *crunchy* and *juicy*, *corn* and *sweet* were sequentially dominant but had comparable citation proportions in early evaluation; *corn* was the most dominant attribute, *fibrous* was the second dominant attribute, and *sweet* was not significantly dominant in TDS, but all three attributes had comparable citations in TCATA in mid evaluation. Similar patterns were observed for *crunchy*, *sweet,* and *juicy* in early evaluation and for *fibrous* and *sweet* in mid and late evaluation of the regular corn. *Smoky* was highly cited throughout the TCATA profiles of the regular and sodium-reduced ham and showed comparable citation proportions to *salty* and *ham* at several timepoints (Figure 3a,b, left). However, during the TDS evaluation this attribute was significantly dominant only for the regular ham and during short time periods (at 45% and 80% of the evaluation time) (Figure 3a, right).

### 3.2. Comparison of Food Product Sensory Attribute Profiles Generated by TDS and TCATA

Differences in temporal sensory profiles of regular and sodium-reduced foods were product specific and depended on the temporal method used for the dynamic sensory characterization. While comparative similarities and differences in sample discrimination by TDS and TCATA are discussed in previous studies [33,34,35,36,37], the current results profile the discriminative ability of TDS and TCATA to compare sensory attribute profiles of regular and sodium-reduced foods. In this section, results of single-intake TCATA and TDS evaluations are described and compared for all of the four study food products.

TDS was more discriminative than TCATA in differentiating regular and sodium reduced foods. Despite greater participant consensus in selecting perceived attributes in TCATA than selecting the dominant attributes in TDS (greater percentages of choices), TDS detected significant differences between regular and sodium-reduced products for a greater number of attributes (Figure 1c, Figure 2c, Figure 3c and Figure 4c). The exception was the cream of mushroom soup for which TCATA detected significant differences between the regular and sodium-reduced soup for a smaller number of attributes but for longer periods of time than TDS; *mushroom* and *cream flavor* were more noticeable in the regular soup for TDS but not TCATA profiles (Figure 4c).

Sodium-reduced products were less characterized by *salty* than their regular counterparts in both TDS and TCATA evaluations of chips and soup, and in TDS but not TCATA evaluation of ham. Specifically, *salty* was highly cited during TCATA evaluation and significantly dominant for the majority of the evaluation time in TDS for the regular chips and soup, but not for sodium-reduced products (Figure 1 and Figure 4). *Salty* was the most cited and significantly dominant attribute in both the regular and sodium-reduced ham for the majority of the evaluation time; the significant differences between the regular and sodium-reduced hams were observed in TDS but not TCATA evaluation of ham (Figure 3). Similarly, regular chips and ham were perceived to be saltier than the sodium-reduced products in a previous study using a 3-point Rate-All-That-Apply scale [56].

In addition to *salty*, other sensory attributes were significantly different between the regular and sodium-reduced products. Greater dominance rates and citation proportions were observed for *dry* in the sodium-reduced chips (Figure 1c), *metallic* or *bitter* in the sodium-reduced ham (Figure 3c), and *thick*, *creamy, sweet,* and *starchy* in the sodium-reduced soup (Figure 4c).

There were many significant attribute differences between the regular and sodium-reduced ham in TDS, whereas *bitter* was the only attribute for which significant differences were found in the TCATA evaluation (Figure 3c). *Bitter* showed greater dominance rates, close to 40% of the evaluation time and greater citation proportions within 55% and 80% of evaluation time in the sodium-reduced than regular ham. *Metallic* was more noticeable in the sodium-reduced than the regular ham in late TDS evaluation, as reflected by the higher dominance rate observed in the TDS difference curve within 65% and 80% of standardized evaluation time. The partial replacement of sodium chloride with salt replacer potassium chloride in the current sodium-reduced ham (Table 1) enhanced the temporal perception of *metallic* and *bitter* tastes, confirming previous reports that potassium chloride induces *metallic* and *bitter* off-taste in processed meats [9,57,58]. The flavor of sodium-reduced ham was described as *metallic* in late TDS evaluation and as *metallic* and *bitter* in late TCATA evaluation, highlighting the information-rich dynamic description of the sensory characteristics of products by TCATA.

In contrast to the other study foods, *salty* was not a dominant attribute of regular corn in either the TDS or the TCATA evaluation. Although *salty* was not significantly dominant in TDS (area under significant level in Figure 2a,b, left), TDS and TCATA significant difference curves (Figure 2c) show greater dominance rates/citation proportions of *salty* in the sodium-reduced corn than in the regular formulation. Higher dominance rates were observed for *sweet* at several timepoints for the regular corn than the sodium-reduced corn (Figure 2, left). The influence of perceptual interactions of salty taste with other flavours contributes to sensory changes in sodium-reduced food products [13,59]. The increased perception of *salty* taste in the sodium reduced canned corn in both TDS and TCATA evaluations can be explained by the taste-taste interaction between *sweet* and *salty*. Saltiness at moderate intensities may enhance sweetness, and sweetness at moderate intensities suppresses salty taste [59]. The greater concentration of sodium chloride in the regular corn may enhance its sweet taste and subsequently mask salty taste perception, thus contributing to greater dominance rates of *sweet* in the TDS profile and smaller dominance rates/citation proportions of *salty* in the TDS/TCATA profile of the regular corn. For sodium-reduced food products in which sweetness is a desirable attribute, a decrease in sweet taste or an increase in salty taste could contribute to consumer preference changes through a change in the expected product temporal profiles.

For chip evaluations, except for *salty* throughout evaluation time and *dry* at specific timepoints, the other sensory attributes showed similar proportion citations between regular and sodium-reduced chips in TCATA profiles, whereas the TDS difference curves revealed significant differences in the dominance rate of *crispy*, *greasy*, *potato*, *sweet* and *umami* at specific timepoints (Figure 1c). *Crispy* showed the highest citation proportion/dominance rate for both regular and sodium-reduced chips in early evaluation. However, the sodium-reduced potato chips showed significantly higher dominance rates for *crispy* during a few seconds at the beginning of TDS evaluation but not for TCATA. On the other hand, *dry*, which showed lower dominance rates/citation proportions than *crispy*, significantly discriminated between the potato chips both in TDS and TCATA for longer periods of time. *Potato* showed the highest dominance rate in the sodium-reduced chips from about 22% standardized time onwards but, was not significantly dominant in the regular sample until about 40% standardized time and reached the highest dominance rate from mid-evaluation onwards.

In contrast to results from the present work, TCATA was reported to provide a more [33,34,35,36] or comparable [37] informative discrimination compared to TDS. The opposing observations of this study may be the result of differences in panel performance (trained panelists, experienced or inexperienced consumers), differences in product categories, the size of the differences between samples, and study objectives [16,29]. Some previous studies involved trained panelists [33,35,36] and others involved consumers [33,37], in which commercial samples of different brands or experimental research samples with tailored ingredient levels were differentiated by both TDS and TCATA. Differences among those samples would be larger than differences between the commercial regular and sodium-reduced formulations of the paired products of the same brand used in this study, differences which are minimized to be acceptable to consumers.

### 3.3. Multiple Intake Evaluation of Chips and Soup

Dynamic sensory product profiles elicited by TCATA and TDS were compared over multiple intakes for potato chips and cream of mushroom soup.

**Figure 5:**
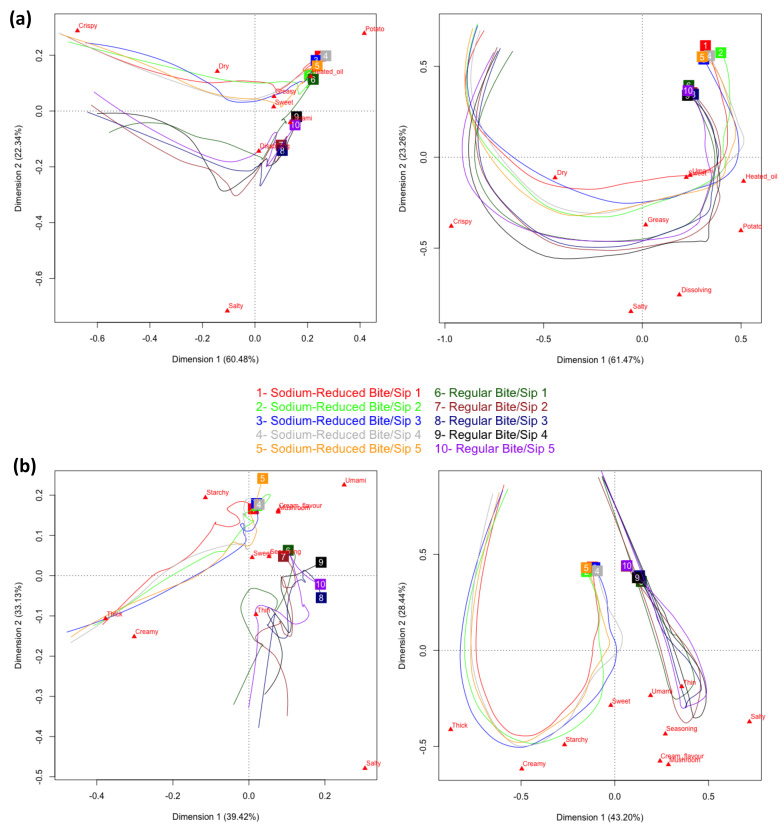
Product trajectories for Principal Component Analysis on time-standardized TDS (left) and TCATA (right) data for multi-intake evaluations of regular and sodium-reduced chips (**a**) and soup (**b**); labeled at the respective trajectory endpoints.

Product trajectories for regular and sodium-reduced chips showed parallel paths along the first dimension of the PCA map for both TDS (60.48%) and TCATA (61.47%) (Figure 5a). In both PCA maps, the first dimension was mainly associated with *crispy* in opposition to *heated oil* and *potato.* Both potato chips were characterized by *crispy* in early evaluation and by *heated oil* and *potato* in late evaluation (Figures S1 and S2). The second dimension of the PCA map for both TDS (22.34%) and TCATA (23.26%) was associated with *salty*, the attribute which most differentiated the regular and sodium-reduced chips. Sodium-reduced chips showed lower citation proportions/dominance rates for *salty* throughout evaluations. The second dimension of the PCA map from the multiple intake TDS evaluation was associated with *crispy* and *potato* in opposition to *salt*. As reflected by the TDS difference curves across intakes, *crispy* and *potato* reached higher dominance rates in the sodium-reduced chips compared to the regular chips (Figure S1).

The difference in sensory attribute profiles between the regular and sodium-reduced products was greater for mushroom soup than for potato chips, as identified by the different directions of the regular and sodium-reduced soup trajectories on the PCA maps for TDS and TCATA (Figure 5b). The first dimension of both PCA maps, with *thick* and *creamy* on the left and *salty* on the right, differentiated the regular and sodium-reduced soups. The sodium-reduced soup showed greater dominance rates/citation proportions of *thick* in early evaluation, whereas the regular soup showed greater dominance rates/citation proportions of *salty* throughout evaluations (Figures S3 and S4). In multiple intakes, *umami* was elicited late in the evaluation of both the sodium-reduced and the regular soup, as observed in the upper right quadrant of the TDS PCA map.

TCATA profiles were more consistent across intakes than TDS profiles; a larger variation among TDS than TCATA trajectories was observed across the multiple intake evaluation for both chips and soup. Attribute profile trajectories were consistent over the multiple intakes for regular and for sodium-reduced products. Patterns of TDS trajectories differed across intakes, with larger variation in the regular than sodium-reduced products (Figure 5, left). The TDS and TCATA curves obtained across the multiple intake evaluation of potato chips and soups samples provided further illustration of the higher variation across intakes for TDS than TCATA (Supplementary Materials, Figures S1–S4).

Differences between the regular and sodium-reduced products were more consistent across intakes for soup than for chips in both TDS and TCATA. Although TDS identified a greater number of attribute differences between regular and sodium-reduced products than TCATA, TDS was less discriminative across sequential intakes (Figure S1). For TCATA, except for *salty*, other attributes differentiating the 2 chips differed across 5 intakes (*dry* for the first and second intake, *crispy, heated oil* and *greasy* for the third intake, *crispy* and *heated oil* for the fourth intake, and *potato* for the fifth intake).

Perceived sensations of a food product are complex due to the interaction among its components. Multi-intake evaluation can help identify the effect of adaptation and build-up of attribute intensity through repeated exposure of the product [60]. Several studies have investigated multi-intake evaluation to assess temporal profiles of food products using TI or TDS [44,48,61], however, this is the first study to profile multi-intake sensory attribute evaluation of sodium-reduced foods using TCATA and TDS. It is reported that multiple intakes may influence sensory perception [62], and changes in TDS profiles were identified across intakes for orange juice with different sweeteners [48]. In the present study, multiple intakes compared to a single intake did not influence TCATA profiles (Figure 5, Figures S2 and S4). For TDS profiles, multiple intakes changed the duration of attribute dominance but not the number of significantly dominant attributes (Figures S1 and S3). This finding aligns with the study of Galmarini et al. [44]; the number of attributes cited did not change from bite to bite in any cheese, and the bite effect on the duration of dominance was observed but not in all of the study samples. Additionally, in the present study, multiple intakes decreased the sample discrimination for TDS but not for TCATA evaluations of chips, whereas multiple intakes did not influence the discrimination between the regular and sodium-reduced soups for either method. The limited effect on the sensory attribute profiles over multiple intakes for both TDS and TCTA may be the consequence of their lack of attribute intensity. This finding was also observed in the study of Antúnez et al. [63] on bread using the descriptive method, Check-All-That-Apply.

### 3.4. Suggestions for Method Selection and Future Research

The results presented here confirm that TDS and TCATA provide complementary information for the dynamic sensory characterization of regular and sodium-reduced foods; TCATA provides a more detailed description of concurrent sensory attributes perceived at a given time while TDS identifies the attributes catching assessors’ attention, as discussed in previous studies [22,33,34,35,36,38]. The dominant attributes, which discriminated more between regular and sodium-reduced foods than the concurrent perceived attributes, may be linked to consumer preference and choice of regular and sodium-reduced products as observed by Ares et al. [34] or C. De Souza et al. [22] for identification of product (dis)liking drivers based on TDS and TCATA profiles. Food reformulation that does not change the TDS sensory attribute profile can be a target for successful development of sodium-reduced foods. Further studies using TDS and TCATA in association with consumer hedonic perception would be beneficial to identify (dis)liking drivers and aid in the selection of the appropriate temporal method to use in sodium-reduced food product development.

This study explored the possibility of using a semi-trained panel for discrimination of regular and sodium-reduced foods. A total of 6 training sessions were used to generate attribute lists and familiarize descriptors and methods to participants for evaluation of 4 product categories by the 2 temporal methods. As an alternative to a consumer panel with a larger number of participants, i.e., 50 consumers or more in previous studies [22,33,47], the current panel of 20 semi-trained participants with triplicate evaluations identified significant differences between the regular and sodium-reduced products for all of the evaluated food items.

The current results of the multi-intake evaluations suggest a thorough consideration of the appropriate method for description of regular and sodium-reduced products; single-intake evaluation could be representative of TCATA profiles over repeated consumption, but not TDS profiles. For TDS profiles, single-intake evaluation could be used for identification of sensory attribute differences between regular and sodium-reduced foods. Multi-intake evaluation will be beneficial in the context of repeated consumption of foods with complex attribute profiles or whose attribute intensities are built up from intake to intake (i.e., sodium-reduced spicy foods and foods containing sweeteners).

## 4. Conclusions

Sodium-reduced prepared food products differed in their temporal sensory attribute profiles compared to their regular counterparts in not only salty taste but other sensory attributes, noticeably *dry* for chips, *sweet* for corn, *bitter* and *metallic* for ham, *thick*, *creamy, sweet,* and *starchy* for soup. Sodium-reduced chips, ham, and soup, but not corn, were less characterized by *salty* than their regular counterparts. TDS and TCATA provided comparable information for the key sensory attributes characterizing and differentiating the regular and sodium-reduced products. TDS was more discriminative than TCATA for single-product intakes, while TCATA generated more consistent profiles across multiple intakes. Multiple intakes changed the duration of attribute dominance but not the number of significantly dominant attributes in TDS profiles. The comparisons across the products and the methods provide a guide to the selection of the most appropriate temporal method for studies of sodium-reduced prepared foods. The current findings and the application of current methods to other food products may support the development of sodium-reduced formulations acceptable to consumers.

## Figures and Tables

**Table 1:** Product and sensory panel testing information for evaluation of potato chips, canned corn, cooked ham and cream of mushroom soup.

Product	Manufacturer Product Information		Sensory Panel Testing Information			
	Brand Name (Ingredients)	Sodium Content	Study Sample Size	Serving Temperature	Swallow Timepoint	Single/Multiple Intake Evaluation
Potato chips	Lay’s Classic Chips, PEPSICO, Canada (potatoes, vegetable oil, salt)	330 mg/50 g	1 chip (approximately 1 g)	Ambient	15 s	Multiple
	Lay’s Lightly Salted Chips with 50% less sodium, PEPSICO, Canada (potatoes, vegetable oil, salt)	160 mg/50 g				
Canned corn	Green Giant Niblet Whole Kernel Corn, B&G Foods Inc., Canada (whole kernel corn, water, salt)	240 mg/125 mL	10 kernels	60 °C	20 s	Single
	Green Giant Niblet Whole Kernel Corn-1/3 Less Salt, B&G Foods Inc., Canada (whole kernel corn, water, salt)	110 mg/125 mL				
Cooked ham	Schneiders Olde Fashioned Ham, Maple Leaf Foods Inc., Canada (ham, water, salt, corn syrup solids, potassium lactate, sodium phosphate, sodium diacetate, sodium erythorbate, sodium nitrite, smoke)	490 mg/55 g	0.3 × 2 × 4 (cm) (approximately 5 g)	Ambient	25 s	Single
	Schneiders Olde Fashioned Ham-25% Less Sodium, Maple Leaf Foods Inc., Canada (pork, water, corn syrup solids, potassium lactate, salt, potassium chloride, potassium phosphate, carrageenan, sodium diacetate, sodium erythorbate, sodium nitrite, smoke)	370 mg/55 g				
Cream of mushroom soup	Campbell’s Cream of Mushroom Soup, Campbell Company of Canada, Canada (water, mushrooms, canola or soybean oil, wheat flour, cream, corn starch, salt, modified milk ingredients, soy protein isolate, monosodium glutamate, tomato paste, spice extract, barley yeast extract, dehydrated garlic)	850 mg/125 mL	10 mL (1 spoon)	60 °C	10 s	Multiple
	Campbell’s Cream of Mushroom Soup-40% Less Salt, Campbell Company of Canada, Canada (water, mushrooms, modified corn starch, cream (milk), canola or soybean oil, wheat flour, butter milk powder, salt, yeast extract, soy protein isolate, white wine, flavour (contains dried onions, chicken), spice extracts)	470 mg/125 mL				

**Table 2:** Sensory attributes and definitions used in TDS and TCATA evaluations of potato chips, canned corn, cooked ham and cream of mushroom soup. An attribute list was prepared for each product.

Modality	Potato Chips	Canned Corn	Cooked Ham	Cream of Mushroom Soup	Definition
Flavour	-	-	Bitter	-	Basic taste associated with caffeine solution
	-	-	Metallic	-	Basic taste associated with various metal flavours
	Salty	Salty	Salty	Salty	Basic taste associated with salt
	Sweet	Sweet	Sweet	Sweet	Basic taste associated with sugar
	Umami	-	Umami	Umami	Basic taste associated with glutamate, salts of amino acids and other molecules called nucleotides
	-	Cooked	-	-	A non-specific flavour associated with the process of heating/cooking
	-	Corn	-	-	Flavour associated to corn
	-	-	-	Cream flavour	A sweet, dairy flavour associated with cream or other high fat dairy products
	-	-	Ham	-	Flavour associated with processed products that contain curing agents (nitrites, sugars, salts)
	Heated oil	-	-	-	Flavour associated with oil heated to a high temperature
	-	-	-	Mushroom	Flavour associated with cooked mushroom
	Potato	-	-	-	Flavour associated with cooked potato
	-	-	-	Seasoning	Flavour associated with seasoning
	-	-	Smoky	-	Dry, dusty flavour of burning wood
	-	Starchy	-	Starchy	Flavour associated with starch
Texture	-	-	Chewy	-	Sensation associated with cohesiveness and to the length of time or the number of chews required to masticate a solid product into a state ready for swallowing
	-	-	-	Creamy	Sensation of creaminess, described as a full, fatty or smooth mouthfeel
	Crispy	-	-	-	Sensation of crispiness, described as the force required to bite while causing a high sound
	-	Crunchy	-	-	Sensation associated with which a sample crumbles, cracks, or shatters
	Dissolving	-	-	-	Sensation caused by moisture absorption and dissolving
	Dry	-	-	-	Sensation of dryness, due to the absence of water or a lack of saliva
	-	Fibrous	Fibrous	-	Sensation refers to long particles oriented in the same direction
	Greasy	-	Greasy	-	Sensation reflects the perception of exuding fat
	-	Juicy	Juicy	-	Sensation caused by higher levels of juices
	-	-	Tender	-	Sensation of tenderness, associated with the ease to chew samples
	-	-	-	Thick	Sensation of thickness, associated with products with a high viscosity
	-	-	-	Thin	Sensation of thinness, associated with products with a low viscosity

## Data Availability

The data presented in this study are available on request from the corresponding author. The data are not publicly available due to ethical reasons.

## Supplementary Materials

The following supporting information can be downloaded at: https://www.mdpi.com/article/10.3390/foods11030457/s1, Figure S1. TDS profiles for regular (left) and sodium-reduced (middle) chips, and significant difference curves (right) of multiple intakes: bite 1 (1), bite 2 (2), bite 3 (3), bite 4 (4) and bite 5 (5) (n = 54). Figure S2. TCATA profiles for regular (left) and sodium-reduced (middle) chips, and significant difference curves (right) of multiple intakes: bite 1 (1), bite 2 (2), bite 3 (3), bite 4 (4) and bite 5 (5) (n = 54). Figure S3. TDS profiles for regular (left) and sodium-reduced (middle) soup, and significant difference curves (right) of multiple intakes: sip 1 (1), sip 2 (2), sip 3 (3), sip 4 (4) and sip 5 (5) (n = 54). Figure S4. TCATA profiles for regular (left) and sodium-reduced (middle) soup, and significant difference curves (right) of multiple intakes: sip 1 (1), sip 2 (2), sip 3 (3), sip 4 (4) and sip 5 (5) (n = 54).
