# Supplementary material for: Temporal Sensory Profiles of Regular and Sodium-Reduced Foods Elicited by Temporal Dominance of Sensations (TDS) and Temporal Check-All-That-Apply (TCATA)
Source: Foods. 2022 Feb 3;11(3):457. doi: 10.3390/foods11030457 (PMC8833930; doi:10.3390/foods11030457)
Supplement: Supplementary file 1 [file foods-11-00457-s001.zip › foods-1558437-supplementary.pdf]

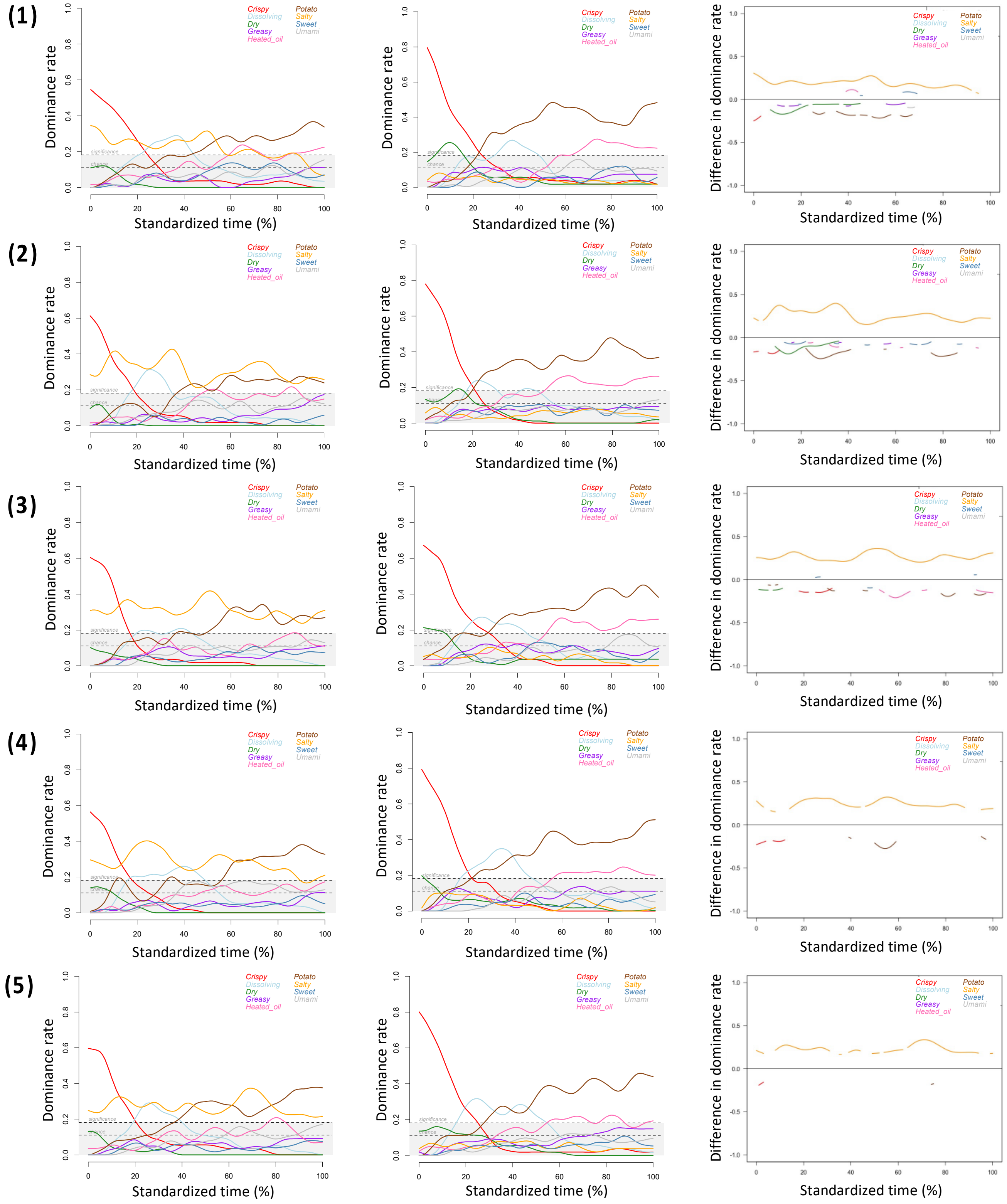

Figure S1. TDS profiles for regular (**left**) and sodium-reduced (**middle**) chips, and significant difference curves (**right**) of multiple intakes: bite 1 (1), bite 2 (2), bite 3 (3), bite 4 (4) and bite 5 (5) (n = 54).

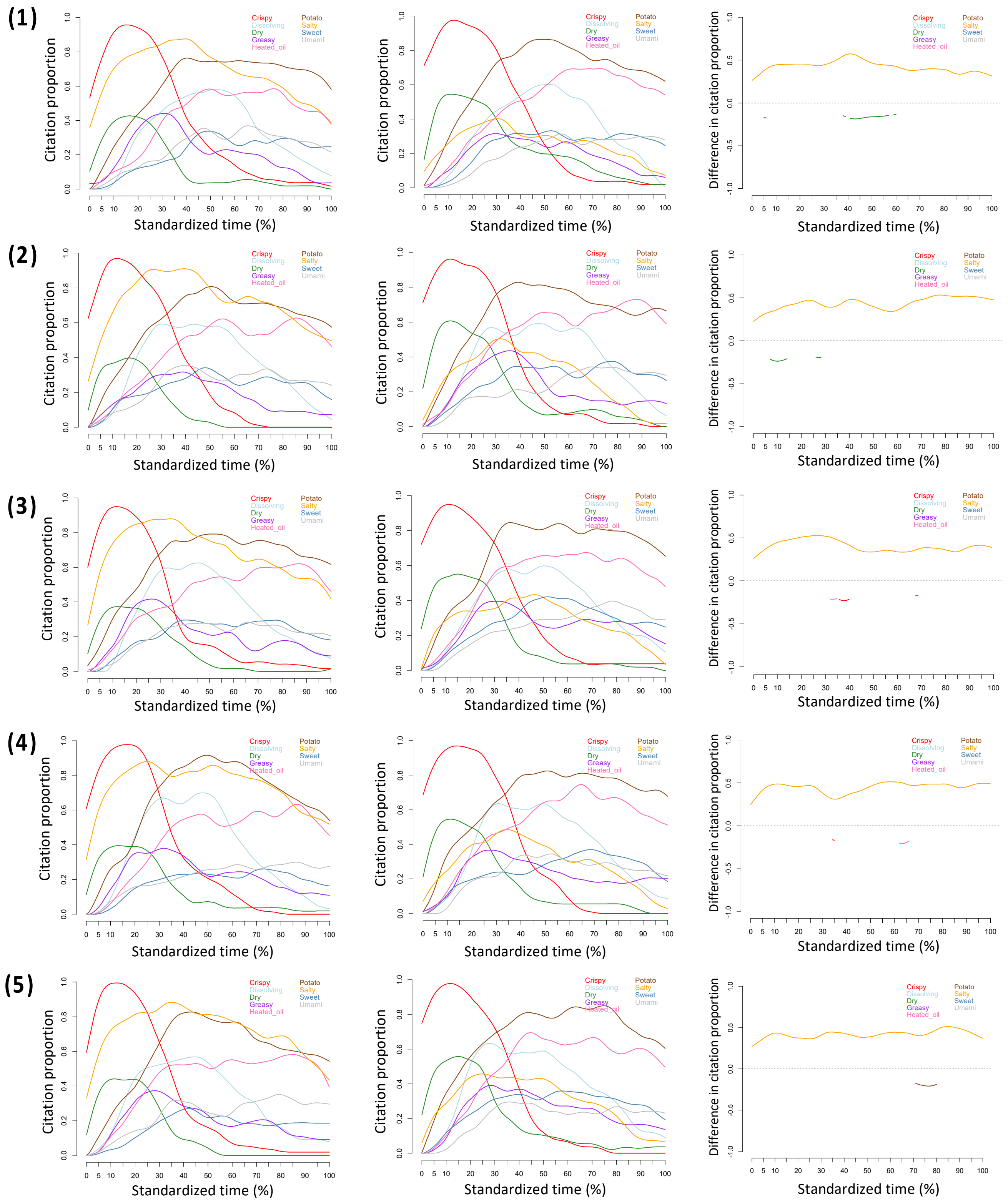

Figure S2. TCATA profiles for regular (**left**) and sodium-reduced (**middle**) chips, and significant difference curves (**right**) of multiple intakes: bite 1 (1), bite 2 (2), bite 3 (3), bite 4 (4) and bite 5 (5) (n = 54).

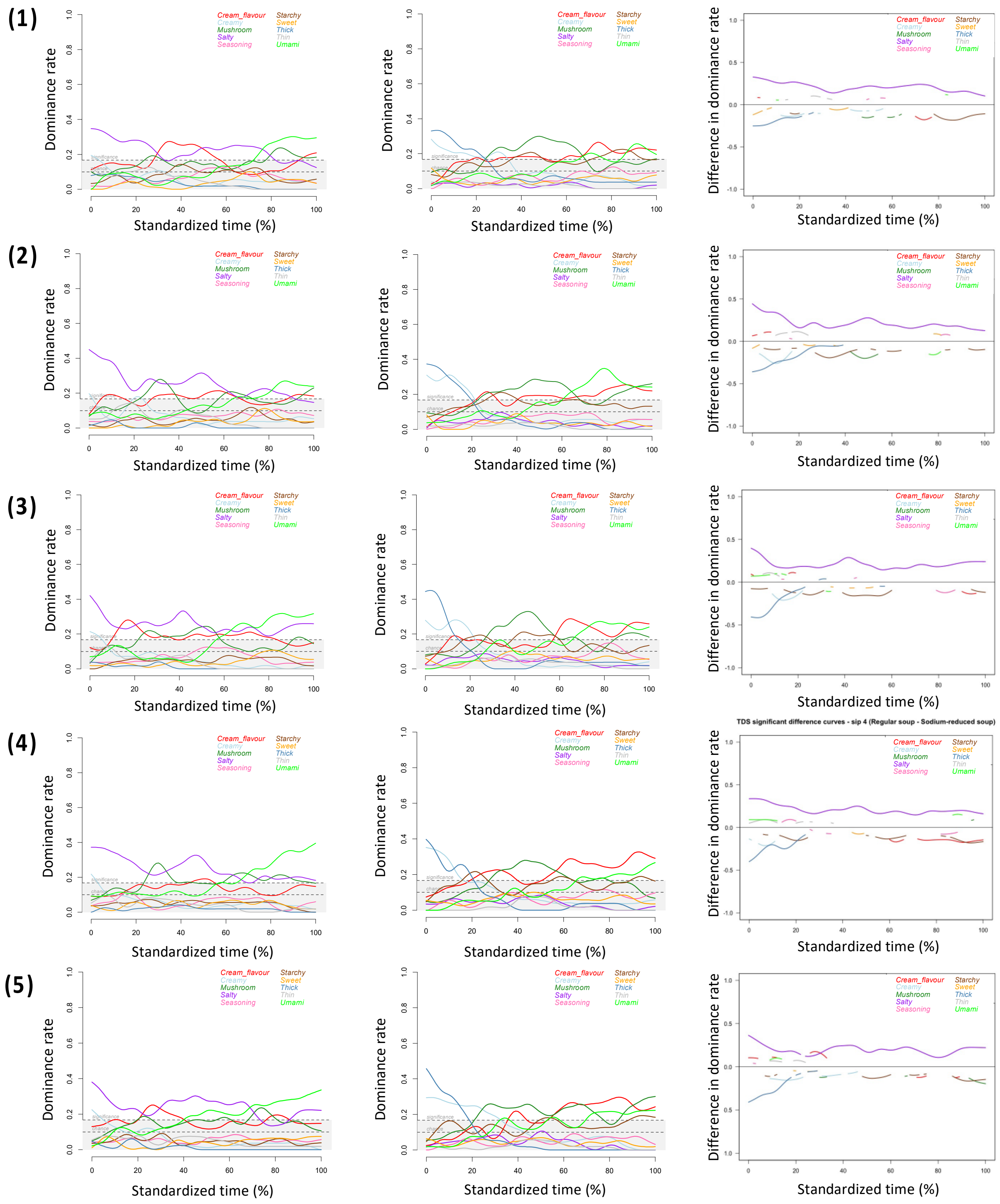

Figure S3. TDS profiles for regular (**left**) and sodium-reduced (**middle**) soup, and significant difference curves (**right**) of multiple intakes: sip 1 (1), sip 2 (2), sip 3 (3), sip 4 (4) and sip 5 (5) (n = 54).

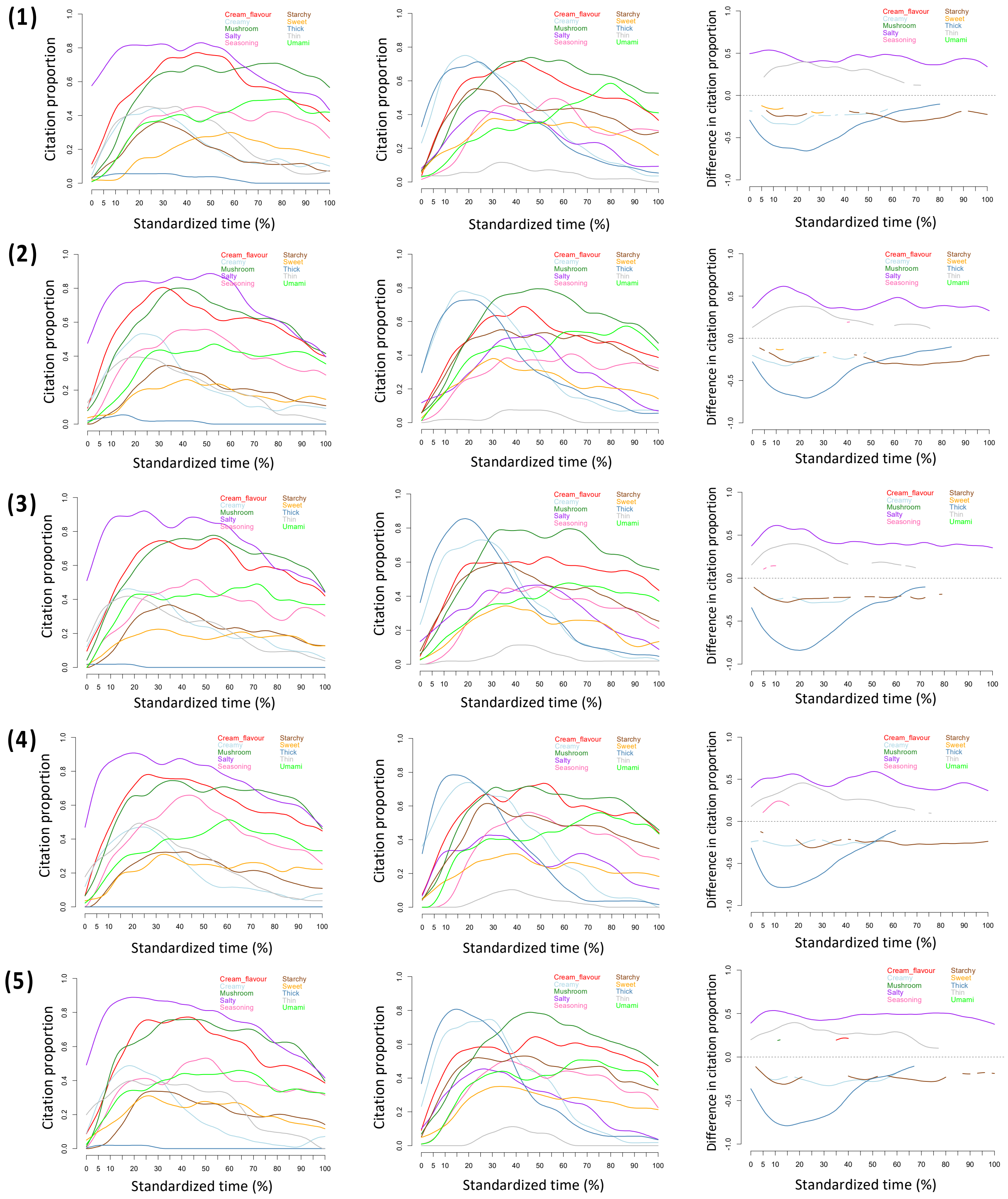

Figure S4. TCATA profiles for regular (**left**) and sodium-reduced (**middle**) soup, and significant difference curves (**right**) of multiple intakes: sip 1 (1), sip 2 (2), sip 3 (3), sip 4 (4) and sip 5 (5) (n = 54).
